# Vasopressin reduces the cumulative epinephrine dose in hypothermic swine with traumatic brain injury

**DOI:** 10.1186/cc12271

**Published:** 2013-03-19

**Authors:** AD Goldberg, MG Kashiouris, L Keenan, A Rabinstein, M Kumar

**Affiliations:** 1 Mayo Clinic, Rochester, MN, USA

## Introduction

Prevention of secondary neurologic injuries is paramount for improved neurologic outcomes after traumatic brain injury (TBI). Evidence suggests that although therapeutic hypothermia (TH) lowers intracranial pressure and attenuates secondary cerebral insults after TBI [1], it also induces hypotension. Brief episodes of mild hypotension in brain-injured patients can trigger secondary injuries, which have been associated with increased mortality in patients with TBI [2]. Vasopressin mitigates hypotension in septic shock and improves coronary perfusion in hypothermic cardiac arrest models [3]. We hypothesized that a low-dose vasopressin infusion may reduce the cumulative epinephrine dose in hypothermic, brain-injured swine.

## Methods

**Figure 1:**
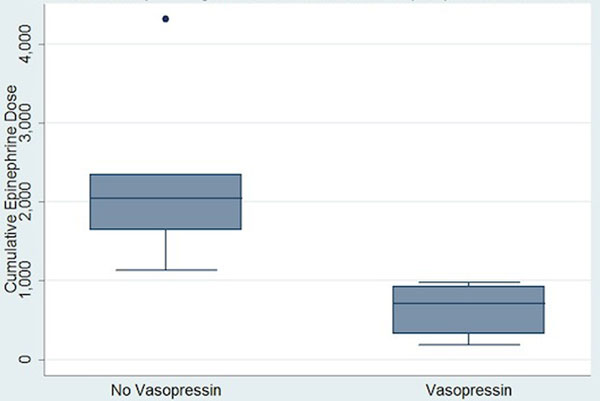
**Effect of vasopressin on cumulative amount of epinephrine administered**.

Six domestic cross-bred pigs were subjected to epinephrine infusion after general anesthesia, standardized TBI and transpulmonary hypothermia (32°C for 48 hours). All animals received the same care, aiming for a mean arterial pressure >60 mmHg. At hour 24, animals received additional vasopressin infusion at 0.04 units/minute. We measured the cumulative epinephrine dose for each animal pre and post vasopressin infusion (Figure 1) and performed a two-sample Wilcoxon rank-sum test, comparing the median cumulative epinephrine doses in the two groups.

## Results

The median cumulative epinephrine dose in the animals that received the vasopressin infusion was 715 mg with a 25th to 75th interquartile range (IQR) of 320 to 930 mg. The median cumulative epinephrine dose in the control group was 2,044 mg (IQR 1,640 to 2,344 mg). This was statistically significant (*P *= 0.003), based on the Wilcoxon rank-sum test.

## Conclusion

A low-dose infusion of vasopressin can significantly reduce vasopressor requirements and improves hemodynamics in hypothermic, brain-injured swine. This hemodynamic stability may improve neurological outcomes.
